# Floristic Richness in a Mediterranean Hotspot: A Journey across Italy

**DOI:** 10.3390/plants13010012

**Published:** 2023-12-19

**Authors:** Marco D’Antraccoli, Lorenzo Peruzzi, Fabio Conti, Gabriele Galasso, Francesco Roma-Marzio, Fabrizio Bartolucci

**Affiliations:** 1 Pisa Botanic Garden and Museum, University of Pisa, Via Ghini 13, 56126 Pisa, Italy; marco.dantraccoli@unipi.it (M.D.); francesco.romamarzio@unipi.it (F.R.-M.); 2 PLANTSEED Lab, Department of Biology, University of Pisa, Via Derna 1, 56126 Pisa, Italy; 3 Floristic Research Center of the Apennine, University of Camerino, Gran Sasso Laga National Park, San Colombo, Barisciano, 67021 L’Aquila, Italy; fabio.conti@unicam.it (F.C.); fabrizio.bartolucci@unicam.it (F.B.); 4 Sezione di Botanica, Museo di Storia Naturale di Milano, Corso Venezia 55, 20121 Milano, Italy; gabriele.galasso@comune.milano.it

**Keywords:** alien species, flora, Mediterranean basin, plant diversity, species–area relationship

## Abstract

Species richness is a fundamental property of biodiversity patterns and is properly expressed by the species–area relationship (SAR), namely the increase in the number of species with the area. Here, we studied and explored the species–area relationship with respect to vascular plant species in Italy and compared vascular plant richness among Italian administrative regions. Concerning the entire vascular flora (native and alien), the best-performing formula is the Arrhenius’ Power function: S = c A^z^. The constants of this function are c = 241.2 and z = 0.281. The best-performing formula concerning just native (c = 245.2 and z = 0.263) and alien (c = 10.1 and z = 0.404) richness is the Power function as well. The floristically richest Italian regions considering the entire flora are Liguria, Friuli Venezia Giulia, and Trentino-Alto Adige, which are also the regions that are richest in alien flora unfortunately. Regions of particular naturalistic interest are Abruzzo, Valle d’Aosta, and Molise, because only these three regions exhibit native floristic richness that is higher than expected, and this is coupled with an alien floristic richness that is lower than expected. On the contrary, four regions (Lombardia, Veneto, Toscana, and Emilia-Romagna) show potentially severe conservation problems due to biological invasions since they experience native floristic richness that is lower than expected, with an alien floristic richness that is higher than expected. This study offers for the first time the ‘c’ and ‘z’ constants specifically calibrated at the national level for Italian vascular flora. The availability of such constants allows the calculation of the number of expected species for a given area to be investigated, providing a robust starting hypothesis for floristic studies.

## 1. Introduction

Our comprehension of plant diversity patterns largely relies on studies based on either local to national floras or distribution maps [1,2]. This is certainly true for the Mediterranean basin, which hosts 20% of the total floristic richness in only 2% of the world’s surface area, characterized by high proportions of endemics and unique species assemblages [3,4,5,6]. Within the Mediterranean basin, the Italian peninsula is among the major centers of species richness for vascular plants, with a high number of endemic taxa [7,8]. This is due to the presence of multiple key areas for plant diversity, acting both as refuge and crossroads, supporting active plant speciation [9]. Italy has a long tradition of national and local floristic studies [10], spanning from the 15th century to the present day. This allowed the accumulation of a great amount of data, which nowadays is the basis for a large spectrum of investigations like systematic studies and deep spatial and temporal analyses of plant diversity patterns [11,12].

Considering species richness as a fundamental property of biodiversity patterns and biotic communities, the theoretical basis and practical applications of the species–area relationship (hereafter SAR), namely the increase in the number of species with respect to area, have been deeply studied and explored [13,14,15]. The first attempts to express this relationship as a mathematical formula were carried out by Arrhenius [16] and Gleason [17] in 1921 and 1922, respectively. Since then, a plethora of mathematical functions have been proposed to describe the relationship between area and species richness [14].

Most studies dealing with SAR in the Mediterranean basin deal with insular systems [18,19,20,21,22,23], while mainland areas have received less attention (however, see [2]). Accordingly, the aims of this study are as follows: (a) implement a species–area relationship with respect to vascular plant species on a national scale (Italy) and (b) compare vascular plant richness among Italian administrative regions, which traditionally are the basic units upon which floristic investigations are carried out in this country.

## 2. Results

### 2.1. Species–Area Relationship (SAR) in Italy

**Figure 1:**
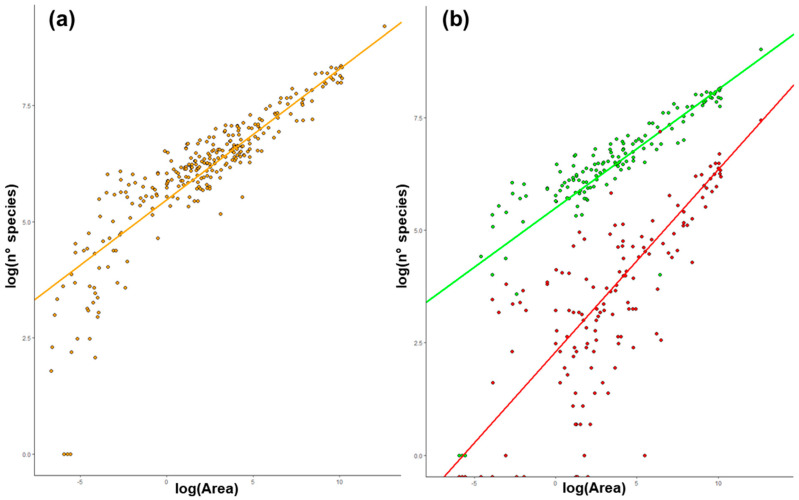
Power function shown in the linearized form (logarithmic space), expressing the relationship between area (*x* axis) and the number of species (*y* axis): (**a**) total flora (i.e., both native and alien species), orange dots and regression line; (**b**) only native (green dots and regression line) and only alien (red dots and regression line) species.

Concerning total species richness, the best-performing SAR formula (Adjusted R^2^ = 0.92; two parameters) is the Power function (Table 1). The constants of the Power function are c = 241.2 and z = 0.281. The best-performing SAR formulae built on native flora (Adjusted R^2^ = 0.91, two parameters; Table 1) and alien flora are their Power functions as well (Adjusted R^2^ = 0.73, two parameters; Table 1). The constants of the Power function are c = 245.2 and z = 0.263 for native flora and c = 10.1 and z = 0.404 for alien flora. SAR functions are graphically represented and expressed in a logarithmic space in Figure 1.

### 2.2. Floristic Richness Comparison among Italian Regions

The floristically richest regions are Liguria, Friuli Venezia Giulia, Trentino-Alto Adige, Abruzzo, and Valle d’Aosta, while Sardegna, Puglia, Sicilia, Emilia-Romagna, and Calabria are the poorest (Table 2).

Considering only native species, the richest regions are Liguria, Friuli Venezia Giulia, Abruzzo, and Valle d’Aosta, while Sardegna, Puglia, Sicilia, and Emilia-Romagna are the poorest (Table 3). Concerning alien species, the richest regions are Liguria, Lombardia, Friuli Venezia Giulia, Trentino-Alto Adige, and Veneto, while Basilicata, Valle d’Aosta, Molise, Calabria, and Puglia are the poorest (Table 4).

**Figure 2:**
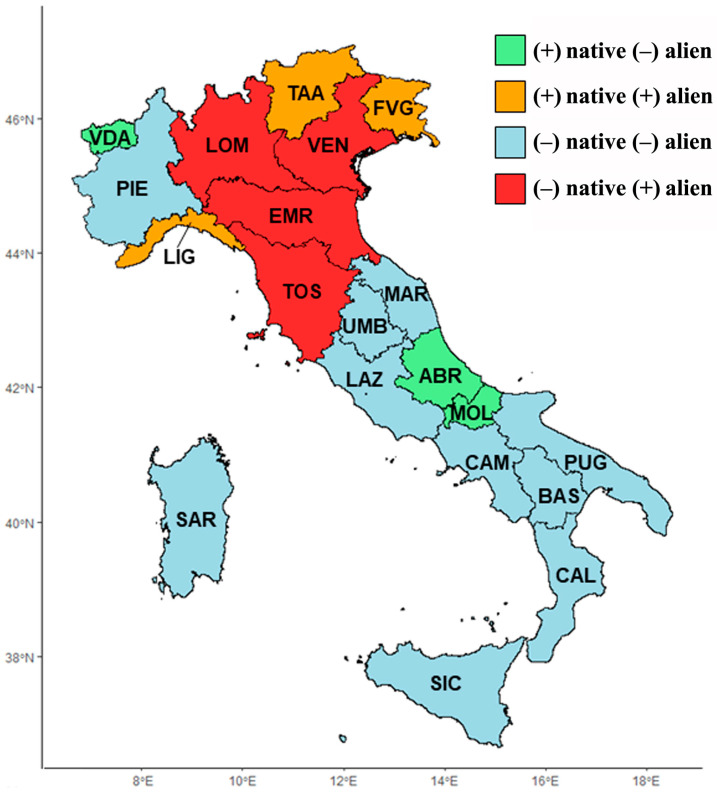
Map showing richness in native and alien species for each administrative region, obtained by contrasting negative vs. positive residuals of the species–area relationship. Acronyms correspond to the following regions: Abruzzo (ABR), Basilicata (BAS), Calabria (CAL), Campania (CAM), Emilia-Romagna (EMR), Friuli Venezia Giulia (FVG), Lazio (LAZ), Liguria (LIG), Lombardia (LOM), Marche (MAR), Molise (MOL), Piemonte (PIE), Puglia (PUG), Sardegna (SAR), Sicilia (SIC), Toscana (TOS), Trentino-Alto Adige (TAA), Umbria (UMB), Valle d’Aosta (VDA), and Veneto (VEN).

Concerning the entire floristic dataset, the residuals of native species richness are positively correlated with those of alien species (Rho = 0.33, *p*-value < 0.0001). Figure 2 summarizes native and alien richness for each administrative region by contrasting positive and negative residuals.

## 3. Discussion

### 3.1. Species Area–Relationship (SAR) in Italy

To the best of our knowledge, this study is the first to offer the constants for a SAR that is specifically calibrated at the national level for Italy. The availability of such constants can allow the calculation of the number of expected species for a given area to be investigated on floristic grounds, as highlighted by [11] (for practical application examples, see [25,26]). Specifically, the expected number of species for a given area can serve as a starting hypothesis and as a reference for inferring the sampling completeness of a floristic survey. In addition, the use of the residuals allows statistically reliable comparisons of floristic richness amongst areas of different extent.

As observed in several previous studies [13,14,15,27], the best-fitting capacity relative to empirical data was achieved via the Power function S = c A^z^ formalized by Arrhenius [16]. The two constants of this function are ‘c’ and ‘z’, which are empirical parameters corresponding to the number of species per unit area (c) and to the increment of the number of species with respect to an increase in area (z).

At the geographical scale of our study, we found that the area extent explained a high proportion of variance relative to the total species richness (R^2^ = 0.92), demonstrating how the area factor is indeed a major driver of floristic richness. A very similar proportion of explained variance was found for native flora (R^2^ = 0.91), while alien flora exhibited a lower value (R^2^ = 0.73). This latter result was somehow expected considering that aliens typically show a more spatially heterogeneous distribution [28], determining a lower variance explained by the species–area relationship [2,29]. This is generally due to the presence of strongly colonized areas and other poorly invaded areas [2].

The ‘c’ value for the entire flora is ~241 species for 1 km^2^, a value that is quite high if compared to the number of species found in the same sampling unit in a tropical lowland rainforest in Colombia (313 species for 1 km^2^), which is the world’s highest value of species richness for this grain [30]. Cowling et al. [3] showed how species richness per standard area varies across Mediterranean climate regions of the world. By comparing their data with our results, we can state that Italy is indeed a species-rich country amongst Mediterranean climate areas.

It has been demonstrated that the ‘z’ parameter can vary with the type of organism [31] and the spatial scale of the sampling [32]. The ‘z’ value for the entire vascular flora (0.281) and native flora (0.263) fell within the typical range expected for vascular plants (0.1–0.40, see [15]). A greater ‘z’ value (0.404) was found instead for the alien portion of the flora, denoting that aliens can increase with area at a higher rate than the whole flora. According to the simulations provided by Blackburn et al. [33], the observed difference in ‘z’ values could be due to the inclusion of casual aliens (i.e., non-established alien species) and insular systems of various sizes in our dataset. The mechanisms underlying the factors shaping these SAR parameters, however, are still far from being fully understood. Despite this, our study exploring empirical data at the country level provides further insights towards a better understanding of this phenomenon.

Eventually, the significant positive correlation between native and alien richness at the national Italian scale is in agreement with the “acceptance hypothesis” [34] and the “rich-get-richer” [35] pattern for explaining biological invasions, as already evidenced at the regional level for Tuscany [2].

### 3.2. Floristic Richness Comparison among Italian Regions

Several authors tried to explore and quantify floristic richness amongst Italian regions in the past, considering both the entire vascular flora [36,37] or woody flora [38]. However, in these studies, SAR was not properly taken into account [36,38] or not considered at all [37].

Considering the total species richness among Italian regions, an increasing gradient from the south to the north can be observed, and this is likely determined by more complex environmental and climatic heterogeneity in northern Italy. Several hypotheses have been formulated to explain latitudinal gradients in species richness, involving water–energy dynamics [39] or history/evolution [40]. A large number of studies confirmed that the relation between climate and species diversity is critical for understanding these patterns, and in particular, current climatic features, such as temperature and precipitation, have been shown to play a great role in explaining the latitudinal patterns of species diversity [41,42]. In addition, a “peninsula effect” could have also contributed to determining this variation pattern, given that species richness is typically known to decrease from the proximal (i.e., northern Italy) to the distal (i.e., southern Italy) areas of a peninsula [43,44,45]. A notable exception to this general pattern is Abruzzo—a central Italian region that is amongst the richest concerning native flora. However, this region extends from the coastline up to the highest peaks of the Apennines (e.g., Gran Sasso, 2912 m a.s.l.), and its higher environmental variability could explain this exception.

By contrasting the SAR results of native (Table 3) vs. alien (Table 4) flora, regions of particular naturalistic interest are Abruzzo, Valle d’Aosta, and Molise (Figure 2). In these three regions, indeed, a native floristic richness that is higher than expected is coupled with an alien floristic richness that is lower than expected. On the contrary, four regions (Lombardia, Veneto, Toscana, and Emilia-Romagna) show potentially severe conservation problems due to biological invasions. In fact, they exhibit native floristic richness that is lower than expected and alien floristic richness that is higher than expected (Figure 2). Interestingly, some Italian regions show many more alien species than expected. This phenomenon could reflect an environmental carrying capacity that is still far from being saturated also in regions that are currently poor in aliens. As the spread of alien species is a highly dynamic and complex system, further steps are needed to understand these patterns.

As a final note, we should bear in mind that species richness alone (*quantity*) does not capture the conservation value of a single species based on rarity, endemicity, or their unique evolutionary history (*quality*) [46]. Indeed, insular Italian regions like Sardinia and Sicily, which are among the poorest concerning floristic richness, show the highest proportion of narrow endemics on the contrary [7,24].

## 4. Materials and Methods

### 4.1. Study Area and Floristic Dataset

**Figure 3:**
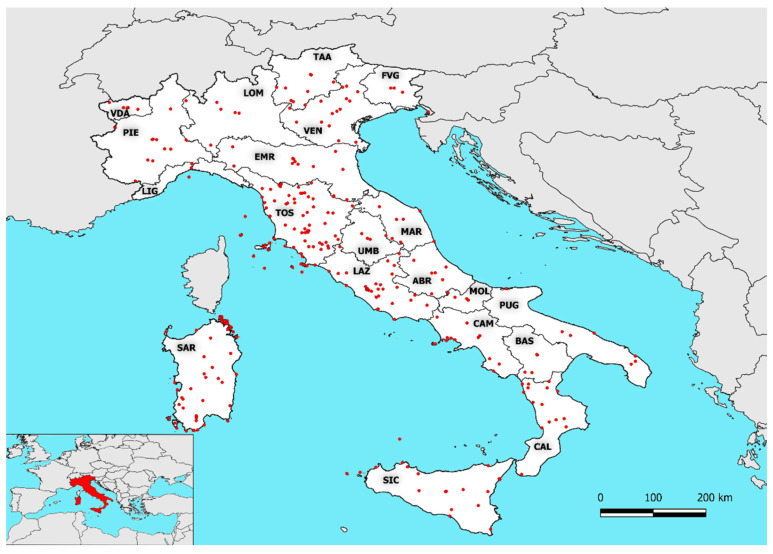
Map of Italy and its twenty administrative regions, showing the localization of the local floras (centroids represented as red points) included in the dataset used to implement the species–area relationship. Acronyms correspond to the following regions: Abruzzo (ABR), Basilicata (BAS), Calabria (CAL), Campania (CAM), Emilia-Romagna (EMR), Friuli Venezia Giulia (FVG), Lazio (LAZ), Liguria (LIG), Lombardia (LOM), Marche (MAR), Molise (MOL), Piemonte (PIE), Puglia (PUG), Sardegna (SAR), Sicilia (SIC), Toscana (TOS), Trentino-Alto Adige (TAA), Umbria (UMB), Valle d’Aosta (VDA), and Veneto (VEN). Acronym positions in the map were arranged to avoid overlap with the sampling points of local floras.

The study area corresponds to Italy (Figure 3), a country lying at the center of the Mediterranean basin, which shows a wide latitudinal extent, representing a long narrow bridge between the temperate and Mediterranean bioclimates [47].

We collated 266 floristic inventories of vascular plants published after 1970, in which the extent of the investigated area was available or clearly inferable, and we added the inventories of the 20 Italian administrative regions and all of Italy, as provided by the Italian checklist [48,49] and following updates [24]. The complete dataset is available in Supplementary Material Table S1. For each flora, we considered the investigated area (minimum value = 0.00123 km^2^; maximum value = 302,068 km^2^; median value = 7.58 km^2^) and both the total number of recorded species and subspecies (referred to as ‘species’ for simplicity) and the number of native species and alien species if available. Our dataset included insular systems and continental areas. We considered both truly native and cryptogenic [48] species as ‘native’, while we considered both established and non-established species (i.e., casual aliens) as ‘alien’. We did not consider taxa which are only cultivated.

### 4.2. Species–Area Relationship (SAR)

To model the relationship between area (A) and species richness (S), we tested the following 14 functions, as provided by the R package ‘sars’ [50]: Asymptotic, Beta-P, Chapman–Richards, Logarithmic, Gompertz, Kobayashi, Linear, Logistic, Monod, Negative Exponential, Power, Rational, Weibull-3, and Weibull-4 (Table 5).

We selected the best-fitting SAR function by considering the following: (1) the explained variance (adjusted R^2^) of the relationship, and the (2) minimum adequate model criteria (i.e., the fewer parameters in the equation, the better). The areas in our datasets comprise a mixed spatial configuration between ‘Type IV’ (areas of varying size, often islands) and ‘Type I’ (nested areas) *sensu* [51]. However, the non-independence of samples does not invalidate the fitting of SAR curves (see [52] for a detailed discussion).

### 4.3. Floristic Richness Comparison among Italian Regions

Residuals in a SAR model reflect the actual floristic richness and compensate for the area effect when comparing floras [2,53]. To allow comparisons among Italian regions, we standardized the residuals as follows.

$$Residuals=\;\frac{n{}^{\circ}\;observed\;species-\;n{}^{\circ}\;expected\;species}{n{}^{\circ}\;expected\;species}\;\times 100$$

After checking the violation of the normal distribution by applying the Shapiro–Wilk test, we assessed the correlation amongst the residuals of native and alien floras via Spearman’s test.

## Figures and Tables

**Table 1:** List of explored functions that describe the species–area relationship in Italy, reporting the main features (shape and the number of parameters) and the explained variance for total and alien species. The asterisk (‘*’) means that it was not possible to compute the parameter statistics of the function.

Function Name	Total Species Adjusted R^2^	Native Species Adjusted R^2^	Alien Species Adjusted R^2^
Asymptotic	0.16	0.24	0.15
Beta-P	*	*	0.60
Chapman–Richards	0.44	0.44	0.41
Logarithmic	0.57	0.64	0.39
Gompertz	0.49	0.58	*
Kobayashi	0.80	0.78	0.68
Linear	0.46	0.49	0.46
Logistic	0.92	0.91	0.73
Monod	0.66	0.63	0.66
Negative Exponential	0.60	0.58	0.65
Power	0.92	0.91	0.73
Rational	0.80	0.81	0.70
Weibull-3	0.92	0.91	0.73
Weibull-4	0.92	0.91	0.73

**Table 2:** List of Italian administrative regions and outputs obtained according to the species–area relationship implemented for the entire flora (native and alien species). The number of species recorded for each region is derived from [24]. The values concerning the number of expected species and the residuals are obtained by applying the Power function, which has proven to be the best–fitting function of the data (see the main text for further details).

Administrative Region	Area (km^2^)	Species Recorded	Species Expected	Residual
Liguria	5418	3574	2701	32.3
Friuli Venezia Giulia	7924	3666	3006	22.0
Trentino-Alto Adige	13,606	4098	3499	17.1
Abruzzo	10,763	3604	3276	10.0
Valle d’Aosta	3263	2507	2343	7.0
Veneto	18,345	4003	3806	5.2
Lombardia	23,844	4242	4097	3.5
Toscana	22,985	4102	4055	1.2
Molise	4461	2525	2558	−1.3
Lazio	17,242	3593	3740	−3.9
Campania	13,590	3298	3498	−5.7
Marche	9344	2946	3148	−6.4
Piemonte	25,387	3836	4169	−8.0
Basilicata	9995	2878	3209	−10.3
Umbria	8456	2709	3061	−11.5
Calabria	15,222	3158	3611	−12.5
Emilia-Romagna	22,510	3418	4031	−15.2
Sicilia	25,711	3262	4184	−22.0
Puglia	19,541	2962	3874	−23.5
Sardegna	24,090	2963	4108	−27.9

**Table 3:** List of the Italian administrative regions and outputs obtained according to the species–area relationship implemented for the native flora. The number of species recorded for each region (native + cryptogenic) is derived from [24]. The values concerning the number of expected species and the residuals are obtained by applying the Power function, which has proven to be the best–fitting function of the data (see the main text for further details).

Administrative Region	Area (km^2^)	Species Recorded	Species Expected	Residual
Liguria	5418	3035	2352	29.0
Friuli Venezia Giulia	7924	2984	2600	14.8
Abruzzo	10,763	3207	2818	13.8
Valle d’Aosta	3263	2299	2059	11.7
Trentino-Alto Adige	13,606	3119	2997	4.1
Molise	4461	2319	2235	3.7
Toscana	22,985	3422	3440	−0.5
Piemonte	25,387	3486	3531	−1.3
Veneto	18,345	3183	3242	−1.8
Lazio	17,242	3045	3190	−4.5
Basilicata	9995	2637	2764	−4.6
Lombardia	23,844	3293	3474	−5.2
Campania	13,590	2829	2996	−5.6
Marche	9344	2528	2715	−6.9
Calabria	15,222	2797	3087	−9.4
Umbria	8456	2371	2645	−10.3
Emilia-Romagna	22,510	2826	3421	−17.4
Sicilia	25,711	2765	3543	−22.0
Puglia	19,541	2562	3296	−22.3
Sardegna	24,090	2330	3483	−33.1

**Table 4:** List of Italian administrative regions and outputs obtained according to the species–area relationship implemented for the alien flora. The number of species recorded for each region (casual aliens + naturalized aliens + invasive aliens) is derived from [24]. The values concerning the number of expected species and the residuals are obtained by applying the Power function, which has proven to be the best-fitting function of the data (see the main text for further details).

Administrative Region	Area (km^2^)	Species Recorded	Species Expected	Residual
Liguria	5418	492	326	51.1
Lombardia	23,844	807	593	36.2
Friuli Venezia Giulia	7924	509	380	34.0
Trentino-Alto Adige	13,606	616	472	30.4
Veneto	18,345	656	533	23.1
Toscana	22,985	657	584	12.5
Emilia-Romagna	22,510	585	579	1.0
Lazio	17,242	516	520	−0.7
Marche	9344	400	406	−1.5
Campania	13,590	465	472	−1.5
Piemonte	25,387	560	608	−7.9
Abruzzo	10,763	378	430	−12.0
Sardegna	24,090	520	595	−12.6
Sicilia	25,711	485	611	−20.6
Umbria	8456	306	390	−21.5
Puglia	19,541	390	547	−28.7
Calabria	15,222	352	494	−28.8
Molise	4461	192	301	−36.2
Valle d’Aosta	3263	166	265	−37.4
Basilicata	9995	248	417	−40.5

**Table 5:** Mathematical functions tested to explore the species–area relationship, their shape, parameters (i.e., the number of constants), and formula.

Name	Shape	Parameters	Formula
Asymptotic	convex	3 (c, d, z)	S = d − c × z^A^
Beta-P	sigmoid	4 (c, d, z, f)	S = d × (1 − (1 + (A/c)^z^)^(−f)^)
Chapman–Richards	sigmoid	3 (c, d, z)	S = d × (1 − exp(−z × A)^c^)
Logarithmic	convex	2 (c, z)	S = c + z × log(A)
Gompertz	sigmoid	3 (c, d, z)	S = d × exp(−exp(−z × (A − c)))
Kobayashi	convex	2 (c, z)	S = c × log(1 + A/z)
Linear	linear	2 (c, z)	S = c + z × A
Logistic	sigmoid	3 (c, f, z)	S = c/(f + A^(−z)^)
Monod	convex	2 (c, d)	S = d/(1 + c × A^(−1)^)
Negative Exponential	convex	2 (d, z)	S = d × (1 − exp(−z × A))
Power	convex	2 (c, z)	S = c × A^z^
Rational	convex	3 (c, d, z)	S = (c + z × A)/(1 + d × A)
Weibull-3	sigmoid	3 (c, d, z)	S = d × (1 − exp(−c × A^z^))
Weibull-4	sigmoid	4 (c, d, f, z)	S = d × (1 − exp(−c × A^z^))^f^

## Data Availability

All the data used in this study are made available in the Supplementary Materials.

## Supplementary Materials

The following supporting information can be downloaded at: https://www.mdpi.com/article/10.3390/plants13010012/s1, Supplementary Material Table S1: Floristic dataset collated to perform SAR analyses.
